# The novel mineralocorticoid receptor modulator balcinrenone protects against diet-induced cardiac microvascular dysfunction and plasma potassium elevation in mouse models

**DOI:** 10.1371/journal.pone.0341078

**Published:** 2026-02-02

**Authors:** Matthew J. Wolf, Soumaya El Moghrabi, Roberto Palacios-Ramirez, Krister Bamberg, Leigh A. Bradley, Frederick H. Epstein, Judith Hartleib-Geschwindner, Frédéric Jaisser

**Affiliations:** 1 Department of Medicine, University of Virginia, Charlottesville, Virginia, United States of America; 2 INSERM, UMRS 1138, Centre de Recherche des Cordeliers, Sorbonne Université, Université de Paris, Paris, France; 3 Research and Early Development, Cardiovascular, Renal and Metabolism, BioPharmaceuticals R&D, AstraZeneca, Gothenburg, Sweden; 4 Late Development, Cardiovascular, Renal and Metabolism, BioPharmaceuticals R&D, AstraZeneca, Gothenburg, Sweden; 5 Université de Lorraine, INSERM Centre d’Investigations Cliniques-Plurithématique 1433, UMR 1116, CHRU de Nancy, French-Clinical Research, Infrastructure Network (F-CRIN) INI-CRCT, Nancy, France; Kurume University School of Medicine, JAPAN

## Abstract

Overactivation of the mineralocorticoid receptor (MR) promotes tissue remodeling in patients with heart failure (HF) and/or chronic kidney disease (CKD). These patients may benefit from MR antagonists (MRAs); however, MRAs are underutilized, partly due to the risk of hyperkalemia. Balcinrenone is a novel, selective MR modulator that demonstrated renoprotection without an acute effect on urinary electrolyte excretion in preclinical studies, suggesting reduced hyperkalemia risk. Here, we present in vivo and in vitro studies comparing balcinrenone with eplerenone, an approved MRA. Myocardial perfusion reserve (MPR), an indicator of coronary microvascular remodeling, was evaluated in mice with diet-induced HF with preserved ejection fraction (HFpEF). MR target gene expression and markers of cardiac remodeling were evaluated using a clonal cell line of rat cardiomyocytes stably expressing MR (H9C2/MR+), and inflammatory and fibrotic processes were evaluated in primary human cardiac fibroblasts. Potassium (K^+^) homeostasis was evaluated in mice with nephrectomy-induced CKD. In mice with diet-induced HFpEF, 30 mg/kg/day balcinrenone or 100 mg/kg/day eplerenone restored MPR to levels seen in mice without HFpEF. Balcinrenone and eplerenone inhibited aldosterone-induced expression of MR target genes and markers of cardiac remodeling in H9C2/MR+ cells, and excretion of collagen 1 and interleukin-6 in primary human cardiac fibroblasts, in a concentration-dependent manner. An overnight K^+^ challenge in eplerenone-treated mice with nephrectomy-induced CKD yielded a higher plasma K^+^ elevation than that observed in vehicle-treated CKD mice. By contrast, the plasma K^+^ response in balcinrenone-treated mice with CKD was similar to what was observed in vehicle-treated CKD mice. Urinary K^+^ excretion was not affected by balcinrenone or eplerenone treatment, but fecal K^+^ excretion was elevated in CKD mice that were administered balcinrenone versus eplerenone. These results suggest that balcinrenone may be suitable for patients requiring additional cardiorenal protection through MR modulation but are at high risk of hyperkalemia.

## Introduction

The mineralocorticoid receptor (MR), NR3C2, is a nuclear receptor expressed by a broad range of epithelial and non-epithelial cell types, with two physiological ligands in humans: aldosterone and cortisol [1,2]. MR activation in classical aldosterone target tissues, such as kidney collecting duct epithelial cells, regulates salt and fluid homeostasis via sodium (Na^+^) reabsorption and potassium (K^+^) excretion, and has a pivotal role in controlling blood pressure [3]. The MR is involved in regulating microvascular function and its activation in non-classical aldosterone target tissues (e.g., cardiomyocytes, endothelial cells, vascular smooth muscle cells, inflammatory cells, and fibroblasts) induces oxidative stress, vascular inflammation, and fibrosis [4–9]. These mechanisms promote pathophysiological changes in the heart, vasculature, and kidney; the consequences of which include cardiac remodeling and heart failure with reduced (HFrEF) as well as preserved (HFpEF) ejection fractions [4,5,7,10], microvascular remodeling (impairment of the coronary vascular bed) [7,11], and the development and accelerated progression of chronic kidney disease (CKD) [6,7,9,12].

Preclinical studies have shown that the deleterious effects of MR overactivation can be mitigated by cell type–specific MR knockout [7]. MR knockout in cardiomyocytes, for example, has been shown to alleviate inflammation and cardiac fibrosis [13] and preserve cardiac function [14]. In humans, MR antagonists (MRAs) provide vital cardiorenal protection for patients with HF and/or CKD [5,6,10,12]. As such, the MRAs spironolactone and eplerenone are recommended for the management of HF [15]. However, spironolactone and eplerenone are frequently underutilized in clinical practice, partly due to the risk of hyperkalemia [16]. Finerenone, a novel MRA, is recommended for the management of CKD in patients with type 2 diabetes who are at high risk of disease progression and cardiovascular events; however, due to the residual risk of hyperkalemia, selecting individuals with consistently normal serum K^+^ levels and regular monitoring of serum K^+^ is still advised [17].

Balcinrenone (AZD9977) is a novel selective MR modulator that is currently in clinical testing. In preclinical models, balcinrenone provided renoprotection without an acute effect on urinary electrolyte excretion, which is suggestive of a reduced risk of hyperkalemia compared with MRAs [18]. Here, we report the results of studies that compared the impact of increasing doses of either balcinrenone or eplerenone on in vivo and in vitro models of cardiorenal disease. The impact on coronary microvascular function in the context of HFpEF was evaluated in mice with diet-induced obesity, comprising impaired myocardial perfusion reserve (MPR; the maximum myocardial blood flow in response to stress [19]) but without obstructive coronary artery disease. The impact on aldosterone-induced expression of MR target genes and markers of cardiac remodeling were assessed in a rat cardiomyocyte cell line with stable MR overexpression (H9C2/MR+), and the impact on aldosterone-induced inflammatory and fibrotic processes was assessed in primary human cardiac fibroblasts. The impact of balcinrenone or eplerenone treatment on K^+^ homeostasis in the context of renal failure, including plasma K^+^ levels and urinary and fecal K^+^ excretion, was evaluated in mice with nephrectomy-induced CKD.

## Materials and methods

### Evaluation of MPR in mice with diet-induced HFpEF

#### Animals and treatment.

The evaluation of MPR in mice was conducted in accordance with protocols that conformed to the Declaration of Helsinki and the National Research Council’s Guide for the Care and Use of Laboratory Animals [20], and was approved by the Animal Care and Use Committee at the University of Virginia (Charlottesville, VA, USA; protocol number 4080 [Wolf]). All efforts were made to minimize suffering to animals.

Ninety-six male C57/B6J mice were purchased from The Jackson Laboratory (Bar Harbor, ME, USA), housed in cages (five mice per cage) with a standard 12-hour light cycle and maintained at 22–25°C, and allowed to acclimatize for 7 days. Six mice provided redundancy in the event of unforeseen problems. At 6 weeks of age, 30 mice were allocated to receive a regular diet (n = 15) (irradiated Teklad^™^ LM-485 [7912]; Envigo, East Millstone, NJ, USA) or a high-fat diet (HFD; n = 15) comprising 60% of calories derived from fat (D12492; Research Diets, Inc., New Brunswick, NJ, USA) for 24 weeks. Sixty mice were allocated to receive an HFD containing admixtures of 10, 30, or 100 mg/kg/day of eplerenone (Molekula Ltd, Newcastle Upon Tyne, UK; n = 10 per dose) or balcinrenone (AstraZeneca, Gothenburg, Sweden; n = 10 per dose). The numbers of mice were determined based on power calculations and prior experiments [21]. Mice were randomized to each treatment group, and the individuals performing and interpreting the magnetic resonance imaging (MRI) scans were blinded to the treatment group.

Individual body weights of the mice were recorded weekly. Food consumption was measured weekly by subtracting the weight of the remaining (uneaten) food from the weight of the food provided. Daily consumption for each mouse was calculated by dividing the food consumed by the number of animals per cage (5) and number of days between weighing (7).

Following 21–23 weeks on their respective diets, all mice were sacrificed after 24 weeks using CO_2_ to effect. Sacrifice of animals at the end of the experiment was pre-determined to obtain organ tissues for cardiac MRI and histology analysis.

#### Cardiac MRI analysis.

Cardiac MRI experimental details have been described previously [21].

Cardiac MRI was performed using a 7T ClinScan system with a 30–35-mm diameter birdcage radio frequency coil (Bruker, Ettlingen, Germany). An indwelling tail vein catheter was inserted to deliver 0.1 mmol/kg of gadolinium-diethylenetriamine pentaacetate acid (Magnevist^®^; Bayer, Berlin, Germany) and 0.1 μg/g of the pharmacological stress agent regadenoson (Lexiscan^®^; Astellas Pharma US Inc., Northbrook, IL, USA) during image acquisition. Body temperature was maintained at 36 ± 0.5°C and anesthesia was maintained using 1.0% to 1.25% isoflurane in oxygen. Localizer imaging was performed to select a mid-ventricular short-axis slice and rest perfusion was imaged using a compressed sensing–accelerated dual-contrast first-pass sequence [22]. Two slices were acquired: one to sample arterial input function (AIF) and a second to sample tissue function (TF). Imaging parameters included the following: echo time/repetition time = 1.2/2.1 ms; field of view = 25.6 × 18 mm^2^; matrix = 128 × 74; phase file of view = 72%; percent sampling = 80%; image resolution = 200 × 250 μm^2^; flip angle = 150°; slice thickness = 1 mm; AIF saturation delay = 15 ms; TF saturation delay = 57 ms; AIF acceleration rate = 6; TF acceleration rate = 4; AIF acquisition time = 25 ms/image; TF acquisition time = 36 ms/image. Thereafter, baseline left ventricular structure and function were assessed using a black-blood cine MRI sequence as previously described [23]. Six to eight short-axis slices were acquired covering the entire left ventricle from base to apex. Imaging parameters included the following: echo time/repetition time = 1.9/4.4 ms; temporal resolution = 4.4 ms; slice thickness = 1 mm; image resolution = 200 μm. Thereafter, regadenoson was injected intravenously and, 10 min later, the first-pass MRI was repeated.

#### Analysis of MRI images.

For perfusion images, image reconstruction and analysis were performed using MATLAB (MathWorks, Inc., Natick, CA, USA). Compressed sensing–accelerated first-pass perfusion images were reconstructed using the Block LOw-rank Sparsity with Motion-guidance (BLOSM) method to compensate for the effects of motion [24]. Perfusion analysis was based on Fermi function deconvolution, as described previously [22]. Using Fermi function deconvolution, rest and stress perfusion were quantified for each mouse at each time point. MPR for each mouse at each time point was calculated as the ratio of stress (regadenoson) perfusion to rest perfusion. Average MPR at each time point was calculated as the mean MPR for all animals at each time point. Black-blood cine images were analyzed using Segment software (Medviso, Lund, Sweden). Specifically, end-diastolic and end-systolic frames were identified and, thereafter, the endocardial and epicardial contours were drawn on these frames for all slices.

#### Histology.

Hearts were excised and fixed in 10% Neutral Buffered Formalin (Thermo Scientific, Inc., Waltham, MA, USA) for a minimum of 4 hours prior to embedding in paraffin. Sections of 10-µm thickness were prepared in short-axis orientation by microtome, with eight sections per glass slide. Five blood vessels of similar size were selected per animal. For quantification of fibrosis, paraffin embedded 10-µm thick short-axis sections were stained with Masson’s Trichrome as previously described [25–27].

### Evaluation of cardiovascular biomarkers in vitro

#### H9C2/MR+ cells.

H9C2/MR+ cells were cultivated as previously described [28], stimulated with 1 nM aldosterone (Sigma Aldrich, Lyon, France) or vehicle for 24 hours, followed by the addition of balcinrenone or eplerenone to final concentrations of 0.1, 0.5, 2.5, or 12.5 µM. We assessed expression levels of six genes previously shown to be regulated by MR in this model and in vivo, as well as several markers of cardiac damage or inflammation: *Sgk-1*, *Ngal*, *Pai-1*, *Adamts1*, *Rgs-2*, and *Serpina3* [28–30].

#### Human cardiac fibroblasts.

Primary human cardiac fibroblasts (PromoCell, Heidelberg, Germany) were cultured in Fibroblast Growth Medium 3 (PromoCell, Heidelberg, Germany) according to the manufacturer’s instruction and used between passages five and seven. Cells were stimulated for 24 hours with or without 10 nM aldosterone, and balcinrenone or eplerenone was added to final concentrations of 0.5, 2.5, or 12.5 µM. Collagen 1 and interleukin-6 (IL-6) concentrations in the supernatant were measured using an enzyme-linked immunosorbent assay (R&D Systems GmbH, Wiesbaden, Germany), which was performed according to the manufacturer’s instructions.

#### Quantitative reverse transcription polymerase chain reaction (PCR) analysis.

H9C2/MR+ cells and human cardiac fibroblasts were lysed using TRIzol^TM^ reagent (Invitrogen, Carlsbad, CA, USA) ahead of RNA extraction. Complementary DNAs were obtained using the M-MLV Reverse Transcriptase kit (Invitrogen, Carlsbad, CA, USA). Quantitative reverse transcription PCR was performed as previously described [31] and reactions were performed in duplicate.

### Evaluation of K^+^ homeostasis in mice with nephrectomy-induced CKD

#### Animals and treatment.

K^+^ homeostasis was evaluated in male C57BL/6J mice, aged 12 weeks, that were purchased from Janvier Labs (Le Genest-Saint-Isle, France), and housed in cages (five mice per cage) with a standard 12-hour light cycle and maintained at 22–25°C. The mice were kept at the Centre d’Explorations Fonctionnelles of the Cordeliers Research Center (Paris, France; agreement B75-06-12). All experiments were performed according to the European Union Directive 2010/63/EU on the protection of animals used for scientific purposes, with approval from the ethics committee “Charles Darwin” of the Sorbonne Université (Paris, France; authorization number APAFIS#3472−20 16031618383911 v3). All efforts were made to minimize suffering to animals.

Twenty-four mice had CKD that was induced under anesthesia through 5/6 nephrectomy. Two poles of the left kidney were removed when mice were 6 weeks of age, and nephrectomy of the right kidney was performed 1 week later; buprenorphine (0.5 mg/kg) was administered before, during, and 24 hours after surgery to mitigate pain. Twenty-four mice underwent sham surgery and received similar procedures that left both kidneys intact. All mice were fed a standard laboratory diet containing 154.3 µmol/g of K^+^ (Safe^®^ Diets, Augy, France) for 4 weeks to allow the manifestation of CKD in mice that received a nephrectomy. Plasma creatinine and urea were then measured. Sham-operated mice and mice with CKD were randomized into groups according to plasma creatinine levels to present homogeneous creatinine levels before starting the K^+^ challenges.

For subsequent experiments, mice were maintained on the standard laboratory diet for a further 5 days. During this time, three groups of eight mice (with CKD and sham operated) received parallel treatment via oral gavage with vehicle (0.5% hydroxypropyl methylcellulose, 0.1% Tween80, and 5% Solutol HS15), eplerenone, or balcinrenone; eplerenone and balcinrenone were administered at doses of 100 mg/kg, twice daily for 4 days, then once on day 5. Overnight K^+^ challenge (between 6 PM on day 4 and 9 AM on day 5) was performed by adding 1.2% (159 mM) potassium chloride to the drinking water.

#### Physiological measurements.

Plasma K^+^ levels were measured on day 5 (4 hours after last administration of vehicle, eplerenone, or balcinrenone) following tail blood retrieval on anesthetized mice using an enterprise point-of-care portable pH/blood-gas analyzer (Siemens Healthcare GmbH, Erlangen, Germany). Tissue, urine, and feces samples were collected after placing the mice in metabolic cages (Tecniplast, Décines-Charpieu, Italy). Urinary K^+^ level was determined by flame photometry (IL943; Instrumentation Laboratory, Ramsey, MN, USA). Plasma and urinary creatinine were analyzed using a Konelab 20i chemistry analyzer (Thermo Scientific, Inc., Waltham, MA, USA). When required, stools were retrieved on day 4 (i.e., before K^+^ challenge) and K^+^ content was measured using flame photometry.

The plasma exposures of balcinrenone and eplerenone relative to their respective MR half-maximal inhibitory concentration values were similar (**S1 Table**).

### Statistical analysis

MPR data are presented as means ± standard error (SE) values. MPR data were evaluated using a Bayesian analysis with broad (uninformative) priors, given the expected limited effect size on MPR reduction with the HFD; analyses were adjusted by using rest values as predictor variables (analysis of variance; ANOVA). Histology data were analyzed by ANOVA followed by Tukey’s multiple comparisons test.

For the evaluation of cardiovascular biomarkers, one-way ANOVA statistical testing was used to compare paired groups of independent samples. ANOVA with Bonferroni adjustment for post hoc tests was used for multiple comparisons.

K^+^ homeostasis data are presented as means (± SE). Data were analyzed using one-way ANOVA followed by a Tukey multiple comparisons post hoc test (GraphPad Software Inc., Boston, MA, USA). The effect of eplerenone (and lack of effect of balcinrenone) on plasma K^+^ elevation after overnight K^+^ challenge in mice with nephrectomy-induced CKD was confirmed in a meta-analysis of five experiments, each including an average of eight animals per treatment group. For each experiment, between-group differences were estimated using a mixed-effect model, with mouse as the random effect since this was a within-group design. Mean between-group differences and their SEs were input into a random effect meta-analysis model (metafor package in R) to determine average effect sizes. *P*-values < 0.05 were statistically significant.

## Results

### Balcinrenone improves cardiac function in mice with diet-induced HFpEF

**Fig 1:**
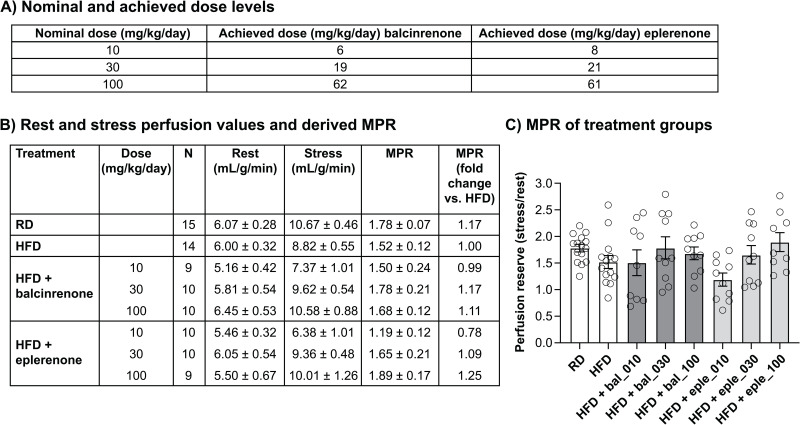
MPR analysis in mice with diet-induced HFpEF: (A) nominal and achieved doses of balcinrenone and eplerenone, (B) rest and stress perfusion values and derived MPR, and (C) MPR of treatment groups. **(A)** The amount of food consumed during the main study was monitored weekly and used to calculate the average doses of balcinrenone and eplerenone that each mouse received. **(B)** Myocardial perfusion before (rest) or after (stress) administration of 0.1 μg/g regadenoson. **(C)** MPR was calculated as: stress perfusion in mL/g/min divided by rest perfusion in mL/g/min, in mice after 20 weeks on the respective diets. Data are presented as means ± standard error. N = 14 or 15 for vehicle groups, N = 9 or 10 for treatment groups. bal, balcinrenone; eple, eplerenone; HFD, high-fat diet; MPR, myocardial perfusion reserve; RD, regular diet.

Evaluation of cardiac function (MPR) was performed using the diet-induced HFpEF model in mice. An initial dose-finding study established that adding 0.08, 0.24, and 0.8 grams of balcinrenone or eplerenone per kilogram of food yielded nominal doses of 10, 30, and 100 mg/kg/day, respectively. The amount of food consumed during the main study was monitored weekly (**S1 Fig**) and used to calculate the average doses that each mouse received (**Fig 1A**), which were within 30% of the nominal doses (for consistency, dose-effect graphs are labeled with the nominal doses).

Three mice that were allocated to receive the HFD alone or with 10 mg/kg/day of balcinrenone or 100 mg/kg/day of eplerenone (n = 1 each) were excluded from the MPR study due to difficulties encountered while placing repetitive intravenous lines or poor quality of stress perfusion images that were caused by motion artifacts at high heart rates.

MPR calculated as the ratio of myocardial perfusion after stress (regadenoson) over perfusion at rest is shown in **Fig 1B and 1C**. Mice that were fed a regular diet had an MPR of 1.78 ± 0.07, and the MPR for the HFD group was 1.52 ± 0.12. Feeding with the HFD plus 10, 30, and 100 mg/kg/day balcinrenone yielded MPR values of 1.50 ± 0.24, 1.78 ± 0.21, and 1.68 ± 0.12, respectively. An HFD plus doses of 10, 30, and 100 mg/kg/day eplerenone yielded MPR values of 1.19 ± 0.12, 1.65 ± 0.21, and 1.89 ± 0.17, respectively.

When compared with mice on an HFD, mice on a regular diet had a 1.17-fold higher MPR. Doses of 10, 30, and 100 mg/kg/day balcinrenone caused 0.99-, 1.17-, and 1.11-fold higher MPRs, respectively, and doses of 10, 30, and 100 mg/kg/day eplerenone caused 0.78-, 1.09-, and 1.25-fold higher MPRs versus mice on HFD without treatment. Bayesian analysis showed, with high probability, that the HFD decreased MPR, and balcinrenone dosing increased MPR to levels that were seen in mice on a regular diet. Eplerenone dosing also increased MPR, but the effect size is smaller than for balcinrenone.

**Fig 2:**
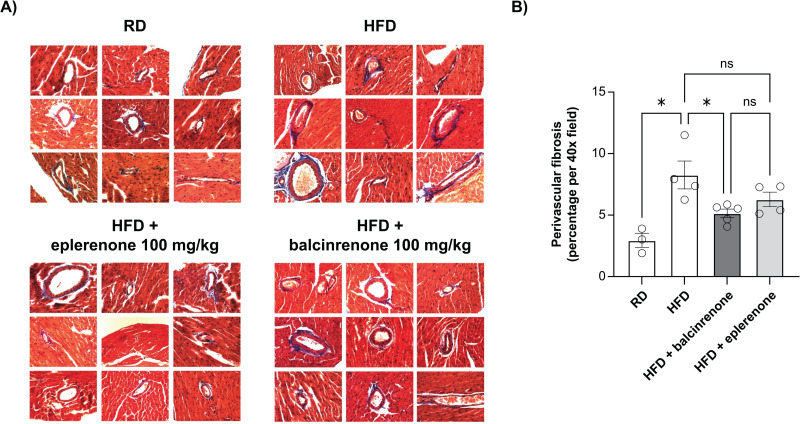
Balcinrenone and eplerenone attenuate cardiac perivascular fibrosis in mice with diet-induced HFpEF. Cardiac tissue samples from mice fed an RD, HFD, or HFD with 100 mg/kg/day balcinrenone or eplerenone were examined for structural changes by histology. **(A)** Representative images of cardiac slides stained with Masson’s Trichrome (three to five hearts per group and two to eight slides per heart). **(B)** Quantification of histology using ImageJ color deconvolution and the blue channel to set thresholds. Data are presented as means ± standard error. *P < 0.05. HFD, high-fat diet; ns, not significant; RD, regular diet.

Cardiac tissue samples from mice that were fed a regular diet, HFD, or HFD with 100 mg/kg/day doses of balcinrenone or eplerenone were examined for structural changes by histology. Increased perivascular fibrosis was observed in mice that were fed an HFD compared with those receiving a regular diet (*P* < 0.05), which was attenuated in mice that were fed an HFD containing balcinrenone (*P* < 0.05 vs. HFD) (**Fig 2**).

### Balcinrenone and eplerenone inhibit expression of MR target genes in H9C2/MR+ cells

**Fig 3:**
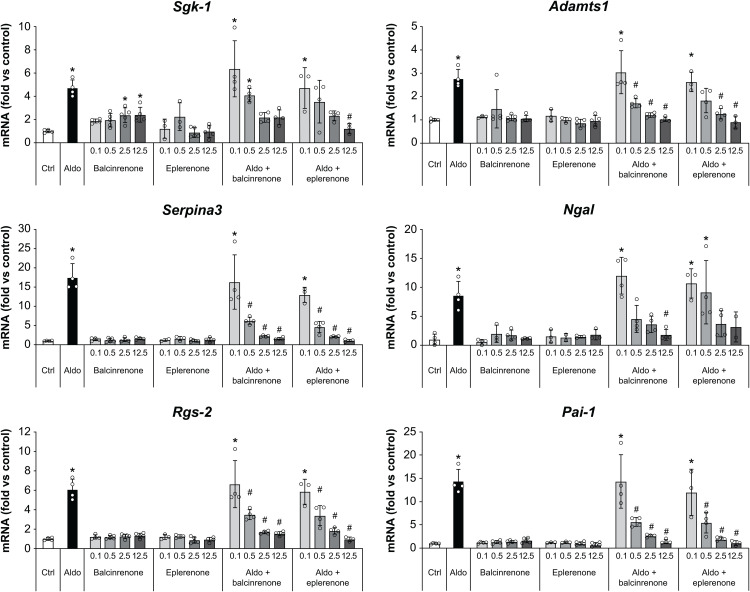
Balcinrenone and eplerenone inhibit aldosterone-induced expression of MR target genes and markers of cardiac remodeling in H9C2/MR+ cells. H9C2/MR+ cells were stimulated with 1 nM aldosterone or vehicle for 24 hours, followed by the addition of balcinrenone or eplerenone to final concentrations of 0.1, 0.5, 2.5, or 12.5 µM. Quantitative reverse transcription polymerase chain reaction was performed to assess gene expression. Data are presented as means ± standard deviations. *P < 0.05 versus control; ^#^*P* < 0.05 versus 1 nM aldosterone. Aldo, aldosterone; Ctrl, control; MR, mineralocorticoid receptor; mRNA, messenger RNA.

The effects of balcinrenone and eplerenone on MR target genes and several markers of cardiac damage or inflammation (*Sgk-1*, *Ngal*, *Pai-1*, *Adamts1*, *Rgs-2*, and *Serpina3* [28–30]) were examined in H9C2/MR+ cells—a rat cardiomyocyte cell line with stable MR overexpression. Stimulation with 1 nM aldosterone induced gene expression (**Fig 3**).

Balcinrenone and eplerenone inhibited aldosterone-induced gene expression in a concentration-dependent manner with similar potencies and efficacies (**Fig 3**). When tested in the absence of aldosterone, neither balcinrenone nor eplerenone affected the expression of *Pai-1*, *Ngal*, *Adamts1*, *Rgs-2*, or *Serpina3*. However, 2.5 and 12.5 µM balcinrenone caused a partial upregulation of *Sgk-1* that corresponded to 37% of the expression levels that were achieved with 1 nM aldosterone. Correspondingly, the inhibition of aldosterone-mediated upregulation of *Sgk-1* plateaued at a slightly higher level (32% of the aldosterone signal) than that observed with eplerenone, which completely suppressed *Sgk-1* upregulation. No partial upregulation of *Sgk-1* was observed with eplerenone in the absence of aldosterone (**Fig 3**).

### Balcinrenone and eplerenone inhibit inflammatory and fibrotic processes in cardiac fibroblasts

**Fig 4:**
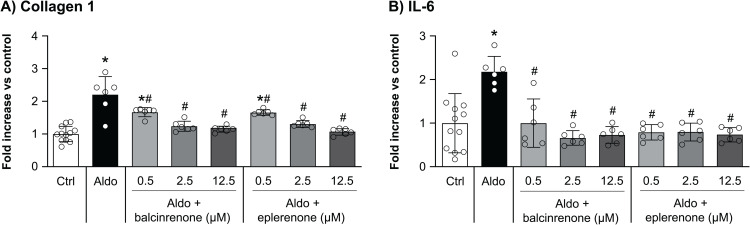
Balcinrenone and eplerenone inhibit (A) fibrotic (collagen 1) and (B) inflammatory (IL-6) processes in primary human cardiac fibroblasts. Primary human cardiac fibroblasts were stimulated for 24 hours with or without 10 nM aldosterone, and balcinrenone or eplerenone added to final concentrations of 0.5, 2.5, or 12.5 µM. **(A)** Collagen 1 and **(B)** IL-6 concentrations in the supernatant were measured using an enzyme-linked immunosorbent assay. Data are presented as means ± standard deviations. *P < 0.05 versus control; ^#^*P* < 0.05 versus 10 nM aldosterone. Aldo, aldosterone; Ctrl, control; IL-6, interleukin-6.

We used primary human cardiac fibroblasts to confirm that fibrotic and inflammatory processes are regulated by the MR across species. Stimulation with 10 nM aldosterone induced excretion of collagen 1 and IL-6 (**Fig 4**). Aldosterone-induced excretion of collagen 1 was inhibited by balcinrenone and eplerenone in a concentration-dependent manner, with full inhibition achieved with a concentration of 12.5 µM. Full inhibition of aldosterone-induced IL-6 excretion was achieved with a concentration of 0.5 µM balcinrenone or eplerenone.

### Balcinrenone does not elevate plasma K^+^ in mice with CKD

**Fig 5:**
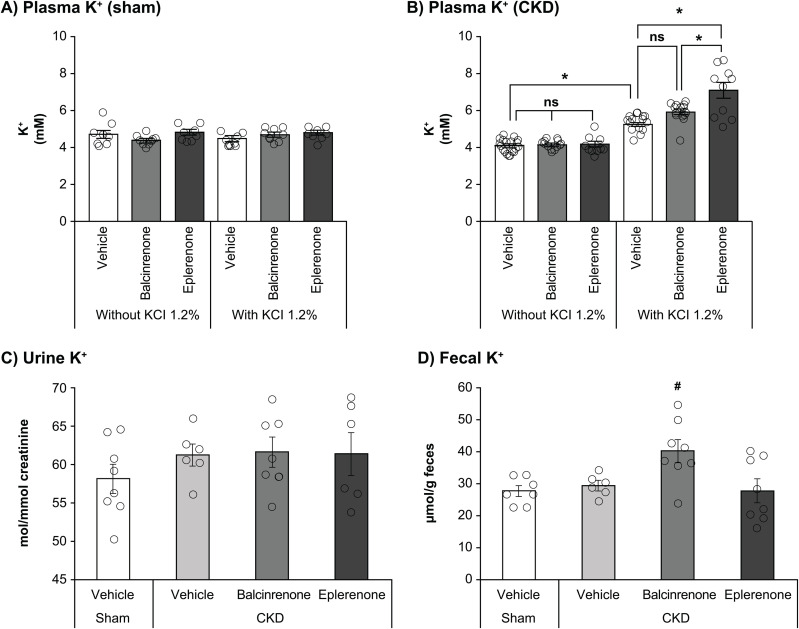
K^+^ homeostasis in the mouse model of nephrectomy-induced CKD: Plasma K^+^ levels in (A) sham-operated mice and (B) mice with nephrectomy-induced CKD before and after K^+^ challenge, (C) creatinine-normalized urinary K^+^ excretion, and (D) fecal K^+^ excretion. Twenty-four mice had CKD induced through 5/6 nephrectomy or had sham surgery. Three groups of eight mice (with CKD and sham operated) received parallel treatment with either vehicle, eplerenone, or balcinrenone; eplerenone and balcinrenone were administered at doses of 100 mg/kg, twice daily for 4 days, then once on day 5. Overnight K^+^ challenge was performed between 6 PM on day 4 and 9 AM on day 5. Plasma K^+^ levels were measured on day 5 (4 hours after last administration of vehicle, eplerenone, or balcinrenone). Urinary and fecal K^+^ levels were measured using flame photometry. K^+^ homeostasis data are presented as means ± standard error. *P < 0.05; ^#^*P* < 0.05 versus vehicle. CKD, chronic kidney disease; K^+^, potassium; KCI, potassium chloride; ns, not significant.

The mouse 5/6 nephrectomy model was used to investigate the effects of balcinrenone and eplerenone on plasma K^+^ in CKD. An overnight K^+^ challenge did not affect plasma K^+^ levels in sham-operated mice administered vehicle, balcinrenone, or eplerenone (**Fig 5A**). In mice with CKD, overnight K^+^ challenge elevated plasma K^+^ levels by ~1.2 mM compared with pre-challenge levels (*P* < 0.05), suggesting that the induced CKD made the mice more susceptible to the K^+^ challenge (**Fig 5B**). In mice with CKD that were administered eplerenone for 5 days, the K^+^ challenge elevated plasma K^+^ levels by ~1.8 mM than what was observed in mice with CKD that were administered vehicle (*P* < 0.05). By contrast, plasma K^+^ after the K^+^ challenge in balcinrenone-treated mice with CKD did not differ from that observed in vehicle-treated mice with CKD, and was ~ 1.2 mM lower than that observed after eplerenone treatment (**Fig 5B**).

The elevation of plasma K^+^ observed in mice with CKD following administration of eplerenone compared with balcinrenone or vehicle, and the lack of treatment effect observed with balcinrenone compared with vehicle following overnight K^+^ challenge, were confirmed in a meta-analysis of five experiments using a random effects model (**S2 Fig**).

### Elevated fecal K^+^ excretion was observed after administration of balcinrenone in mice with CKD

The effects of balcinrenone and eplerenone on urinary and fecal K^+^ excretion were determined in the mouse 5/6 nephrectomy model. Creatinine-normalized urinary K^+^ excretion prior to the overnight K^+^ challenge was slightly elevated in the CKD mice but was unaffected by either balcinrenone or eplerenone treatment (**Fig 5C**). Fecal K^+^ excretion was similar between sham-operated mice and mice with CKD (**Fig 5D**). In mice with CKD, fecal K^+^ excretion was elevated in balcinrenone-treated animals compared with vehicle, but was unaffected following administration of eplerenone (**Fig 5D**).

## Discussion

Patients with moderate-to-advanced CKD or HF with comorbid CKD may benefit from additional cardiorenal protection with MRAs in line with management guidelines [15,17]. However, these patients continue to be sub-optimally dosed or denied treatment with MRAs altogether due to the risk of hyperkalemia [6,16]. Therefore, there is an increasing interest in identifying novel MRAs with a differentiated safety profile. Balcinrenone is a novel selective MR modulator with a differentiated mode of action. Clinical studies in patients with CKD with or without HF have demonstrated beneficial effects of balcinrenone in combination with the sodium-glucose co-transporter 2 inhibitor dapagliflozin versus dapagliflozin alone on urinary albumin-to-creatinine ratio, with only minor increases in serum K^+^ concentrations and a low incidence of hyperkalemia [32,33]. In preclinical studies, balcinrenone has been shown to dose-dependently reduce albuminuria and improve kidney histopathology in a similar manner to eplerenone, but without acute effects on urinary Na^+^/K^+^ ratio [18]; this is in contrast to MRAs that dose-dependently increase the urinary Na^+^/K^+^ ratio [18,34]. In the current study, we extend the preclinical findings to show beneficial effects of balcinrenone on MPR in an in vivo model of HFpEF and a reduced effect on plasma K^+^ in a CKD model.

The current studies aimed to explore cardiac benefits of balcinrenone and eplerenone using in vivo and in vitro models of HFpEF and relevant pathways. Previously, MRAs have been shown to improve coronary microvascular function [11]. In our study, increasing doses of balcinrenone and eplerenone were evaluated in mice with diet-induced HFpEF and reduced MPR [21,35]. This model reflects cardiac microvascular dysfunction but does not show significant changes in other cardiac functional parameters or interstitial fibrosis. Reduced MPR is indicative of poor prognosis [36] and an elevated risk for major adverse cardiovascular events [37] in patients with cardiomyopathy. Balcinrenone restored MPR in mice with diet-induced HFpEF and attenuated perivascular fibrosis with at least similar efficacy as eplerenone. Both balcinrenone and eplerenone were also shown to inhibit aldosterone-induced fibrotic (collagen 1) and inflammatory (IL-6) processes in primary human cardiac fibroblasts with similar efficacy. These findings across different species indicate comparable cardioprotective effects of balcinrenone and eplerenone using in vivo and in vitro models of HFpEF.

We also aimed to evaluate the impact of balcinrenone and eplerenone on the gene expression mediating these MR-activated molecular mechanisms. Aldosterone-induced genes have previously been elucidated in H9C2/MR+ cells [28]. Here, H9C2/MR+ cells were stimulated with aldosterone, and increasing concentrations of balcinrenone and eplerenone were evaluated for their impact on the expression of six genes: *Sgk-1* (involved in cell signaling and electrolyte handling), *Pai-1* and *Adamts1* (involved in extracellular matrix regulation and may potentiate cardiac remodeling, and *Pai-1* also increases collagen deposition and may potentiate cardiac fibrosis), *Rgs-2* (involved in signal transduction and vascular tone regulation), and *Ngal* and *Serpina3* (involved in regulating matrix metalloproteinase) [4,28–30]. Aldosterone-induced expressions of *Sgk-1*, *Ngal*, *Pai-1*, *Adamts1*, *Rgs-2*, and *Serpina3* were inhibited by balcinrenone and eplerenone in a concentration-dependent manner, with similar potencies, in line with previous observations from a reporter gene assay [18]. Preclinical and clinical data suggest that MR blockade can attenuate fibrosis, inflammation, and tissue remodeling, and improve cardiac and vascular function, and may underpin the cardiorenal protection associated with MRAs [38,39]. Notably, collagen 1 and IL-6 have been shown to promote cardiac fibrosis and inflammation in rats [40]. Given that the proinflammatory and profibrotic effects of MR activation may be potentiated by the upregulation of *Pai-1* [28] and *Ngal* [6], the effects of balcinrenone and eplerenone on inhibiting inflammatory (IL-6) and fibrotic (collagen 1) processes may involve inhibition of aldosterone-induced expression of *Pai-1* and *Ngal*.

Impairment of renal K^+^ excretion appears to be a key factor in MRA-mediated hyperkalemia [18]. Previous findings have shown that balcinrenone had no acute effect on urinary electrolyte excretion in rats that were fed a low salt diet to increase endogenous aldosterone levels [18]; this is in contrast to MRAs, which elevate the Na^+^/K^+^ ratio in a dose-dependent manner and hence increase hyperkalemia risk. The present studies aimed to further elucidate the effects of balcinrenone on K^+^ homeostasis. The impact of 100 mg/kg balcinrenone or eplerenone on K^+^ homeostasis was evaluated in mice with nephrectomy-induced CKD that were subjected to overnight K^+^ challenge. K^+^ challenge did not affect plasma K^+^ levels in sham-operated animals, but K^+^ challenge resulted in elevated plasma K^+^ levels in a mouse model of nephrectomy-induced CKD. Importantly, plasma K^+^ levels were further elevated in CKD mice that were administered eplerenone versus vehicle; however, plasma K^+^ levels were not different in CKD mice that were administered balcinrenone versus control and were lower for CKD mice that were administered balcinrenone versus eplerenone. The potential organs involved in this electrolyte response were investigated by analyzing the excretion of K^+^ via urinary and fecal elimination. Previous studies in the 5/6 nephrectomy mouse model of CKD have shown either similar [41] or elevated [42,43] urinary K^+^ excretion compared with control animals. In the present study, urinary K^+^ excretion prior to overnight K^+^ challenge was slightly elevated in CKD mice, but was unaffected by either balcinrenone or eplerenone treatment. Instead, fecal K^+^ excretion was found to be higher in mice that were administered balcinrenone versus eplerenone. This suggests that the colon plays a more extensive role compared with the kidney in mediating electrolyte regulation in this model of CKD.

In conclusion, we report that the novel MR modulator balcinrenone improved cardiac microvascular perfusion and histopathology, with comparable efficacy to eplerenone, but with a reduced and differential effect on K^+^ homeostasis in cardiorenal mouse models. This is relevant, as approved MRAs such as eplerenone and spironolactone are often underutilized, partly due to the risk of hyperkalemia. In a mouse CKD model, mice that were administered balcinrenone and subjected to an acute K^+^ challenge displayed elevated fecal K^+^ excretion compared with mice that were administered eplerenone, in contrast to similar levels of urinary K^+^ excretion. The colon therefore plays a more extensive role in mediating the different effects of balcinrenone and eplerenone on K^+^ homeostasis in this model of CKD. Understanding the exact mechanisms contributing to the differential regulation of electrolyte homeostasis with balcinrenone will require further elucidation. These results, combined with those of previous preclinical studies [18], suggest that balcinrenone may be a suitable alternative for patients with HF and/or CKD who require additional cardiorenal protection but are at high risk for hyperkalemia. Whether these findings translate into cardiorenal benefits for such patients warrants further evaluation in clinical trials.

## Supporting information

S1 Fig MPR analysis in mice with diet-induced HFpEF: (A) weekly body weight, (B) food consumption, and (C) achieved dose level with balcinrenone and eplerenone. (EPS)

S2 Fig Meta-analysis of K^+^ homeostasis showing point estimates and 95% CIs for the treatment effect of eplerenone versus vehicle, and balcinrenone versus vehicle or eplerenone, in mice with nephrectomy-induced CKD. (EPS)

S1 Table Plasma concentrations of balcinrenone or eplerenone after twice-daily dosing of 100 mg/kg in mice with CKD prior to an overnight K^+^ challenge. (DOCX)
