# Supplementary material for: The novel mineralocorticoid receptor modulator balcinrenone protects against diet-induced cardiac microvascular dysfunction and plasma potassium elevation in mouse models
Source: PLoS One. 2026 Feb 2;21(2):e0341078. doi: 10.1371/journal.pone.0341078 (PMC12863481; doi:10.1371/journal.pone.0341078)
Supplement: S1 Table — (DOCX) [file pone.0341078.s003.docx]

**S1 Table. Plasma concentrations of balcinrenone or eplerenone after twice-daily dosing of 100 mg/kg in mice with CKD prior to an overnight K^+^ challenge.**

|  | **Balcinrenone (µM)** | **Eplerenone (µM)** |
| --- | --- | --- |
| **Mean ± SD** | 5.39 ± 1.84 | 6.96 ± 1.78 |

Blood samples following tail blood retrieval on anesthetized mice were analyzed for drug exposures as previously described [1].

CKD, chronic kidney disease; K^+^, potassium; SD, standard deviation.

**Reference**

1. Bamberg K, Johansson U, Edman K, William-Olsson L, Myhre S, Gunnarsson A, et al. Preclinical pharmacology of AZD9977: A novel mineralocorticoid receptor modulator separating organ protection from effects on electrolyte excretion. PLoS One. 2018;13:e0193380. <https://doi.org/10.1371/journal.pone.0193380> PMID: 29474466.
